# Role of Botulinum Toxin in Treatment of Depression: A Review

**DOI:** 10.1192/bjo.2025.10154

**Published:** 2025-06-20

**Authors:** Qutub Jamali, Mahmoud Ali, Doaa Sadek

**Affiliations:** 1 Lancashire and South Cumbria Foundation Trust, Lancashire, United Kingdom; 2 Central North West Foundation Trust, London, United Kingdom

## Abstract

**Aims:** Depressive disorders, as defined by the International Classification of Diseases (11th Revision), involve a depressive mood (feeling sad, irritable, or empty) along with other symptoms affecting a person’s ability to function. Botulinum toxin, used for conditions like migraines and muscle spasms, is being researched as a treatment for depression. The theory is based on facial feedback, where paralysing facial muscles could reduce the brain’s ability to process negative emotions, potentially improving mood. This review aims to explore how botulinum toxin might work in depression treatment and summarize the current research and future directions in this field.

**Methods:** We searched PubMed. Inclusion criteria were paper should discuss depression and botulinum. We excluded papers before 2010 and papers which included botulinum toxin in patient comorbid migraine.

**Results:** Magid et al. reviewed 2 case series, 3 randomized controlled trials (RCTs), and one meta-analysis on botulinum toxin for depression. 29\39 U was injected. They concluded that botulinum toxin was not yet an appropriate treatment.

Kugar and Wollmer examined studies on botulinum toxin’s effects on depression, especially patients with chronic migraine. 29/39 U was injected. Some improvement but called for further research.

Wollmer et al. (2019) analysed 4 RCTs and 3 case series, confirming the efficacy of botulinum toxin in treating depression. They found a lasting effect of about 3 months with a single treatment but recommended further research.

Qian et al. (2020) conducted a systematic review of RCTs and concluded that botulinum toxin might offer a new treatment option for major depressive disorder. However, the effects on depression secondary to other conditions remain unclear.

Danilo et al. (2021) analysed 5 RCTs, finding botulinum toxin more effective than a placebo in treating depression. They noted low risk of bias, with statistically significant results.

Yang Li et al. (2021) reviewed 10 RCTs and confirmed that studies support botulinum toxin as a potential alternative treatment for depression.

Wollmer et al. (2022) evaluated 5 studies and found botulinum toxin effective for patients although the exact mechanisms are still under study.

**Conclusion:** While botulinum toxin has shown potential in treatment for depression, particularly for patients who didn’t respond to antidepressants or who experience side effects, the current evidence remains preliminary. Several studies indicate that botulinum toxin may offer symptomatic relief. However, the overall quality of the evidence is limited by small sample sizes, methodological inconsistencies, and the need for more trials.

